# Respiratory and Gastrointestinal Microbiome Shifts in Livestock Exposed to High-Ammonia Environments: A Systematic Review

**DOI:** 10.3390/vetsci13090952

**Published:** 2026-09-12

**Authors:** Andrei Ungur, Claudiu-Nicusor Ionică, Mircea Coroian

**Affiliations:** 1 Department of Porcine Health Management, Faculty of Veterinary Medicine, University of Agricultural Sciences and Veterinary Medicine of Cluj-Napoca, 400372 Cluj-Napoca, Romania; andrei.ungur@usamvcluj.ro; 2 Department of Animal Nutrition, Faculty of Veterinary Medicine, University of Agricultural Sciences and Veterinary Medicine of Cluj-Napoca, 400372 Cluj-Napoca, Romania; 3 Department of Poultry Management and Pathology, University of Agricultural Sciences and Veterinary Medicine of Cluj-Napoca, 400372 Cluj-Napoca, Romania; mircea.coroian@usamvcluj.ro; 4 Department of Parasitology and Parasitic Diseases, University of Agricultural Sciences and Veterinary Medicine of Cluj-Napoca, 400372 Cluj-Napoca, Romania

**Keywords:** environmental monitoring, host–microbe interactions, sustainable husbandry, functional ecology

## Abstract

Environmental NH_3_ is a major pollutant in intensive livestock production, but its effects on host-associated microbiota remain insufficiently characterized. This systematic review evaluated the impact of environmental NH_3_ exposure on the respiratory and gastrointestinal microbiome of poultry, swine, and cattle. Following PRISMA 2020 guidelines, PubMed, Web of Science, and Scopus were searched, identifying 175 records. After duplicate removal and screening, eight studies were included: five in broiler chickens and three in pigs, while no eligible bovine studies were identified. NH_3_ exposure was associated with alterations in microbial diversity and composition, accompanied by epithelial injury, inflammatory activation, oxidative stress, and impaired production outcomes. Evidence was strongest in broilers, where both respiratory and gastrointestinal microbial communities were affected. In pigs, gastrointestinal microbiome and metabolic alterations were demonstrated, whereas evidence for respiratory microbiome involvement was more limited. Microbiota depletion and transplantation experiments in broilers further indicated that intestinal dysbiosis may contribute to respiratory injury through TLR4/MyD88/NF-κB signaling, supporting a potential gut–lung interaction. Overall, environmental NH_3_ represents an important ecological stressor affecting livestock microbiota, although standardized longitudinal and mechanistic studies, particularly in pigs and cattle, are needed to establish causality and species-specific responses.

## Simple Summary

Ammonia (NH_3_) is a common environmental pollutant in intensive livestock farming that can negatively affect animal health. This systematic review examined how environmental ammonia exposure influences the respiratory and gastrointestinal microbiome of poultry, pigs, and cattle. Eight studies met the inclusion criteria, including five studies in broiler chickens and three in pigs, while no eligible studies were identified in cattle. Overall, ammonia exposure was associated with changes in microbial diversity and composition, together with inflammation, tissue damage, and impaired animal performance. The strongest evidence was found in broilers, where ammonia affected both intestinal and respiratory microbial communities. Some experimental studies also suggested that changes in the intestinal microbiome may contribute to respiratory injury through the gut–lung axis. However, the available evidence remains limited and differs considerably between species. Further controlled and long-term studies, particularly in pigs and cattle, are needed to better understand these relationships and their implications for livestock health and production.

## 1. Introduction

According to United Nations projections, the global population is expected to reach approximately 9.7 billion by 2050, with further increases anticipated in the following decades. This continuous demographic growth is expected to substantially increase the global demand for food, particularly animal-derived products [1,2]. The intensification of modern livestock production systems has led to the widespread use of enclosed animal housing facilities designed to maximize productivity and efficiency [3,4]. However, these intensive rearing environments are frequently associated with the accumulation of airborne pollutants, particularly NH_3_ [5]. NH_3_ is one of the most prevalent gaseous pollutants encountered in intensive livestock systems and represents a major environmental and animal welfare concern worldwide [3,6]. In confined animal housing, NH_3_ is primarily generated through the microbial degradation of nitrogenous waste products excreted by animals, including urea in mammals and uric acid in poultry. Livestock manure therefore constitutes the principal substrate for NH_3_ volatilization in modern production facilities [3,4,5].

In poultry production, NH_3_ generation is strongly associated with the microbial decomposition of uric acid accumulated in litter-based environments. Microbial uricolytic activity converts uric acid into ammonium (NH_4_^+^), which is subsequently transformed into gaseous NH_3_ depending on environmental conditions such as pH, moisture, and temperature [3,5,7].

In swine and dairy systems, NH_3_ mainly originates from the enzymatic hydrolysis of urinary urea mediated by urease-producing microorganisms present in manure and slurry storage systems. This process rapidly converts urea into total ammoniacal nitrogen, which may subsequently volatilize into the air as gaseous NH_3_ [8,9]. Additional contributions arise from the mineralization of undigested proteins and other organic nitrogen compounds present in feces [10,11].

The rate of NH_3_ generation and volatilization is influenced by several environmental and managerial factors, including stocking density, ambient temperature, relative humidity, dietary crude protein content, manure handling practices, and ventilation efficiency [3,12]. The physicochemical characteristics of litter materials also play an important role in NH_3_ volatilization dynamics. Factors such as litter moisture retention capacity, pH, porosity, absorbency, and microbial activity can substantially influence the conversion of ammoniacal nitrogen into gaseous NH_3_. Bedding materials with high moisture accumulation and poor absorbency tend to favor microbial degradation processes and increase NH_3_ release, whereas dry, highly absorbent litter materials may reduce NH_3_ volatilization [3,7]. Elevated temperatures and alkaline conditions favor NH_3_ volatilization, whereas inadequate ventilation promotes the accumulation of gaseous NH_3_ within animal housing facilities [5,6].

Due to the high animal densities and prolonged indoor confinement characteristic of modern intensive farming systems, NH_3_ accumulation has become a persistent challenge across poultry, swine, and cattle operations [3,12]. Consequently, chronic exposure to elevated NH_3_ concentrations is increasingly recognized not only as an environmental issue, but also as a biological stressor capable of impairing animal health, welfare, immune function, and production performance [13,14,15,16].

Air quality is considered a critical component of animal health, welfare, and productivity in intensive livestock production systems. Within enclosed animal housing facilities, the accumulation of airborne contaminants such as NH_3_, carbon dioxide (CO_2_), hydrogen sulfide (H_2_S), particulate matter, dust, and bioaerosols may adversely affect both animals and workers [6,15]. Among these pollutants, NH_3_ is widely recognized as one of the most important indicators of indoor air quality due to its high prevalence and well-documented biological effects in poultry, swine, and cattle production systems [3,12].

Several regulatory agencies and scientific organizations have proposed guideline values or exposure thresholds for NH_3_ concentrations in livestock housing. In poultry production, NH_3_ concentrations below 10 ppm are generally considered optimal for animal welfare and respiratory health, whereas concentrations exceeding 20–25 ppm are commonly associated with impaired air quality and increased risk of adverse health effects [6,15]. Occupational exposure limits established for human workers are typically higher; for example, the Occupational Safety and Health Administration (OSHA) and the National Institute for Occupational Safety and Health (NIOSH) recommend exposure limits of 25 ppm for prolonged occupational exposure [17]. However, these standards are primarily intended for humans and may not adequately reflect the chronic sensitivity of animals continuously exposed to NH_3_ within confined housing systems.

Reported NH_3_ concentrations in livestock facilities vary considerably depending on species, housing design, season, ventilation efficiency, stocking density, and manure management practices. In commercial poultry houses, NH_3_ concentrations frequently range between 10 and 50 ppm, with peak levels occasionally exceeding 70–100 ppm under conditions of poor litter quality or inadequate ventilation [3,18]. Similar NH_3_ concentrations have been reported in swine confinement systems, particularly during colder seasons when ventilation rates are reduced to conserve heat [12]. NH_3_ levels may also exhibit substantial diurnal and seasonal variation associated with fluctuations in ambient temperature, humidity, and microbial activity within manure and litter substrates [11,12].

Chronic exposure to elevated NH_3_ concentrations has been associated with multiple adverse effects on animal health and production performance. Experimental and field studies have demonstrated that prolonged NH_3_ exposure may impair respiratory tract integrity, reduce feed efficiency and growth performance, increase susceptibility to infectious diseases, and negatively affect animal welfare [15,16]. In poultry, NH_3_ exposure has additionally been linked to ocular irritation, tracheal lesions, altered immune responses, and increased predisposition to secondary bacterial infections [3,6]. Beyond animal-related effects, NH_3_ emissions also represent an important environmental and occupational concern due to their contribution to atmospheric pollution, eutrophication, and particulate matter formation [12,13].

Although current air quality standards provide useful reference values for controlling NH_3_ exposure in livestock housing, they may not fully capture the subclinical biological effects associated with long-term exposure to low or moderate NH_3_ concentrations [13,15]. Emerging evidence suggests that environmental stressors within animal housing systems can influence host-associated microbial communities, immune homeostasis, and disease susceptibility [19]. Therefore, the microbiome is increasingly considered a sensitive biological indicator of environmental stress and a potential early-warning marker of impaired animal health.

## 2. Methods

### 2.1. Literature Search Strategy

A systematic literature search was conducted according to the Preferred Reporting Items for Systematic Reviews and Meta-Analyses (PRISMA 2020) guidelines to identify original studies investigating the effects of environmental ammonia exposure on the respiratory and gastrointestinal microbiome of targeted livestock species [20]. Three electronic databases, PubMed, Web of Science Core Collection, and Scopus, were searched independently. The search strategy combined free-text terms related to ammonia exposure, microbiome/microbiota, and the target livestock species (poultry, swine, and cattle) using Boolean operators (AND/OR). Database-specific search syntax was adapted according to the indexing system of each database while maintaining the same conceptual search strategy. The complete database-specific Boolean search strings were as follows. For PubMed, the search strategy was: (ammonia[Title/Abstract] OR NH_3_[Title/Abstract] OR “ammonia exposure”[Title/Abstract] OR “ambient ammonia”[Title/Abstract]) AND (microbiome[Title/Abstract] OR microbiota[Title/Abstract] OR dysbiosis[Title/Abstract] OR pathobiome[Title/Abstract] OR “gut microbiome”[Title/Abstract] OR “intestinal microbiome”[Title/Abstract] OR “respiratory microbiome”[Title/Abstract] OR “airway microbiome”[Title/Abstract]) AND (poultry[Title/Abstract] OR broiler[Title/Abstract] OR chicken[Title/Abstract] OR swine[Title/Abstract] OR pig*[Title/Abstract] OR porcine[Title/Abstract] OR cattle[Title/Abstract] OR bovine[Title/Abstract]) AND (exposure[Title/Abstract] OR environment*[Title/Abstract] OR housing[Title/Abstract] OR barn*[Title/Abstract] OR air[Title/Abstract]). For Web of Science Core Collection, the search strategy was: TS = (ammonia OR NH3 OR “ammonia exposure” OR “ambient ammonia” OR “air ammonia”) AND (microbiome OR microbiota OR dysbiosis OR pathobiome OR “gut microbiome” OR “intestinal microbiome” OR “respiratory microbiome” OR “airway microbiome”) AND (poultry OR broiler* OR chicken* OR swine OR pig* OR porcine OR cattle OR bovine) AND (exposure OR environment* OR housing OR barn* OR air). For Scopus, the search strategy was: TITLE-ABS-KEY(ammonia OR NH3 OR “ammonia exposure” OR “ambient ammonia” OR “air ammonia”) AND (microbiome OR microbiota OR dysbiosis OR pathobiome OR “gut microbiome” OR “intestinal microbiome” OR “respiratory microbiome” OR “airway microbiome”) AND (poultry OR broiler* OR chicken* OR swine OR pig* OR porcine OR cattle OR bovine) AND (exposure OR environment* OR housing OR barn* OR air)**. For PubMed, the search was performed within the Title/Abstract field using the following filters: publication date (last 5 years), English language, full-text availability, and Other Animals. This search yielded 21 records. For Web of Science Core Collection, the search was conducted using the Topic (TS) field in the Advanced Search interface. Filters included publication date (last 5 years), document type (Research Article), English language, and Open Access, resulting in 73 records. For Scopus, the search was performed within the TITLE-ABS-KEY field using the corresponding database-specific syntax. The same filters were applied, including publication date (last 5 years), document type (Research Article), English language, and Open Access, retrieving 81 records.

### 2.2. Selection of Studies

In total, 175 records were identified across the three databases (PubMed, *n* = 21; Web of Science, *n* = 73; Scopus, *n* = 81). Following duplicate removal (*n* = 47), 128 unique records remained and were subjected to title and abstract screening. The database-specific search strategies, together with the applied filters and number of records retrieved, are additionally summarized in Supplementary Table S1.

#### 2.2.1. Title and Abstract Screening

Following the removal of 47 duplicate records, 128 unique articles remained and underwent title and abstract screening according to the predefined eligibility criteria. During this stage, articles were assessed for their relevance to the objective of the review, namely the effects of environmental ammonia exposure on respiratory and gastrointestinal microbiome alterations in livestock species. Of the 128 screened records, 113 articles were excluded, while 15 studies were considered potentially eligible and advanced to the full-text assessment stage. The reasons for exclusion during title and abstract screening were as follows: 56 articles were not related to environmental ammonia exposure, 34 were not relevant to the scope of the review, 12 were not conducted in the target livestock species (poultry, swine, or cattle), 6 did not investigate respiratory or gastrointestinal microbiome alterations, 4 were review articles, and 1 represented an additional duplicate identified during screening publication (Supplementary Table S4). Where more than one exclusion criterion was applicable, the primary reason for exclusion was recorded.

#### 2.2.2. Full Text Screening

**Figure 1:**
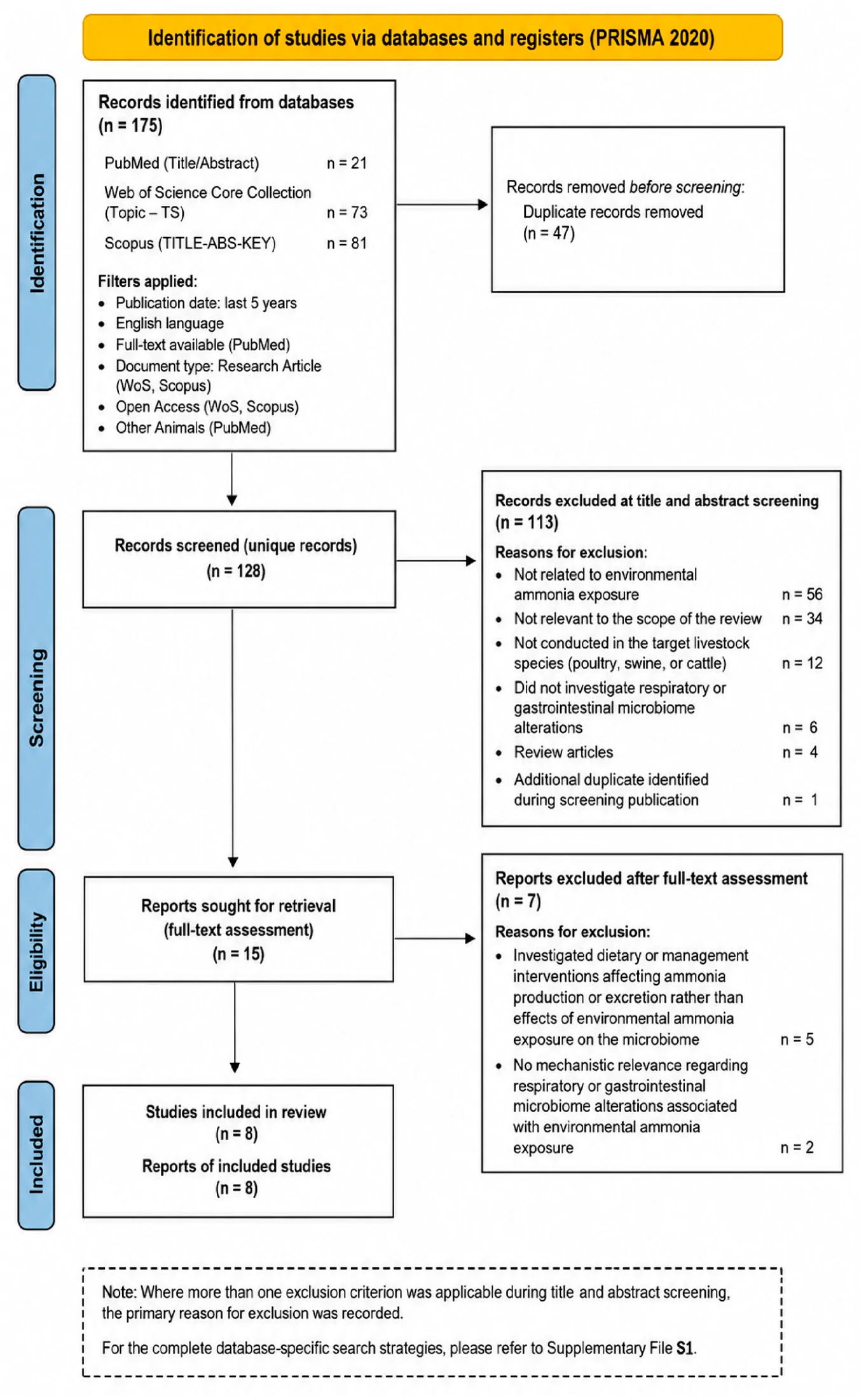
PRISMA Flow diagram.

Following title and abstract screening, 15 articles were considered potentially eligible and underwent full-text assessment against the predefined eligibility criteria. Of these, 8 studies fulfilled the eligibility criteria and were included in the systematic review, while 7 studies were excluded following full-text evaluation. The main reason for exclusion was that the investigated relationship between ammonia and the microbiome did not correspond to the exposure–outcome relationship addressed by the present review. Specifically, five studies investigated dietary or management interventions affecting ammonia production or excretion rather than the effects of environmental ammonia exposure on the host microbiome (Figure 1). The remaining two studies were excluded because they did not provide mechanistic relevance regarding respiratory or gastrointestinal microbiome alterations associated with environmental ammonia exposure (Supplementary Table S5).

#### 2.2.3. Risk of Bias Assessment

The methodological quality of the eight studies included in the systematic review was assessed using the SYRCLE Risk of Bias tool for animal intervention studies (Tables S2 and S3). The assessment considered ten domains: random sequence generation, baseline characteristics, allocation concealment, random housing, blinding of caregivers and investigators, random outcome assessment, blinding of outcome assessment, incomplete outcome data, selective outcome reporting, and other potential sources of bias. Each domain was classified as low, high, or unclear risk of bias, and an overall risk-of-bias judgement was subsequently assigned to each study. Two reviewers (A. Ungur & M. Coroian) independently performed study selection according to predefined eligibility criteria. Generative Artificial Intelligence (GenAI) tools were used to assist in the preparation of this manuscript. Specifically, GenAI was employed to facilitate the final verification and consistency of the PRISMA study selection counts, and to assist with language editing and paraphrasing of portions of the manuscript text. All AI-generated outputs were carefully reviewed, verified, and revised by the authors. The authors take full responsibility for the content of the manuscript. Furthermore, all references, citations, study eligibility decisions, data extraction procedures, and interpretations of the scientific literature were independently checked and validated by the authors to ensure their accuracy and integrity.

#### 2.2.4. Study Selection and Inter-Reviewer Agreement

Study selection was independently performed by two reviewers according to predefined eligibility criteria. At both screening stages, each reviewer independently classified the records as eligible or ineligible according to the predefined criteria, and reasons for exclusion were documented (Tables S4 and S5). Complete agreement was achieved between the two reviewers, with no discrepancies requiring discussion or arbitration. Inter-reviewer agreement was assessed using Cohen’s kappa coefficient (κ). Considering the final eligibility decisions for all 128 records, both reviewers independently classified 8 records as included and 120 as excluded, with no discordant decisions. The observed agreement was 100% (P_o_ = 1.000), while the expected agreement by chance was P_e_ = 0.883. This resulted in a Cohen’s κ of 1.000, indicating perfect inter-reviewer agreement throughout the study-selection process.

## 3. Results

### 3.1. Species-Specific Responses

**Figure 2:**
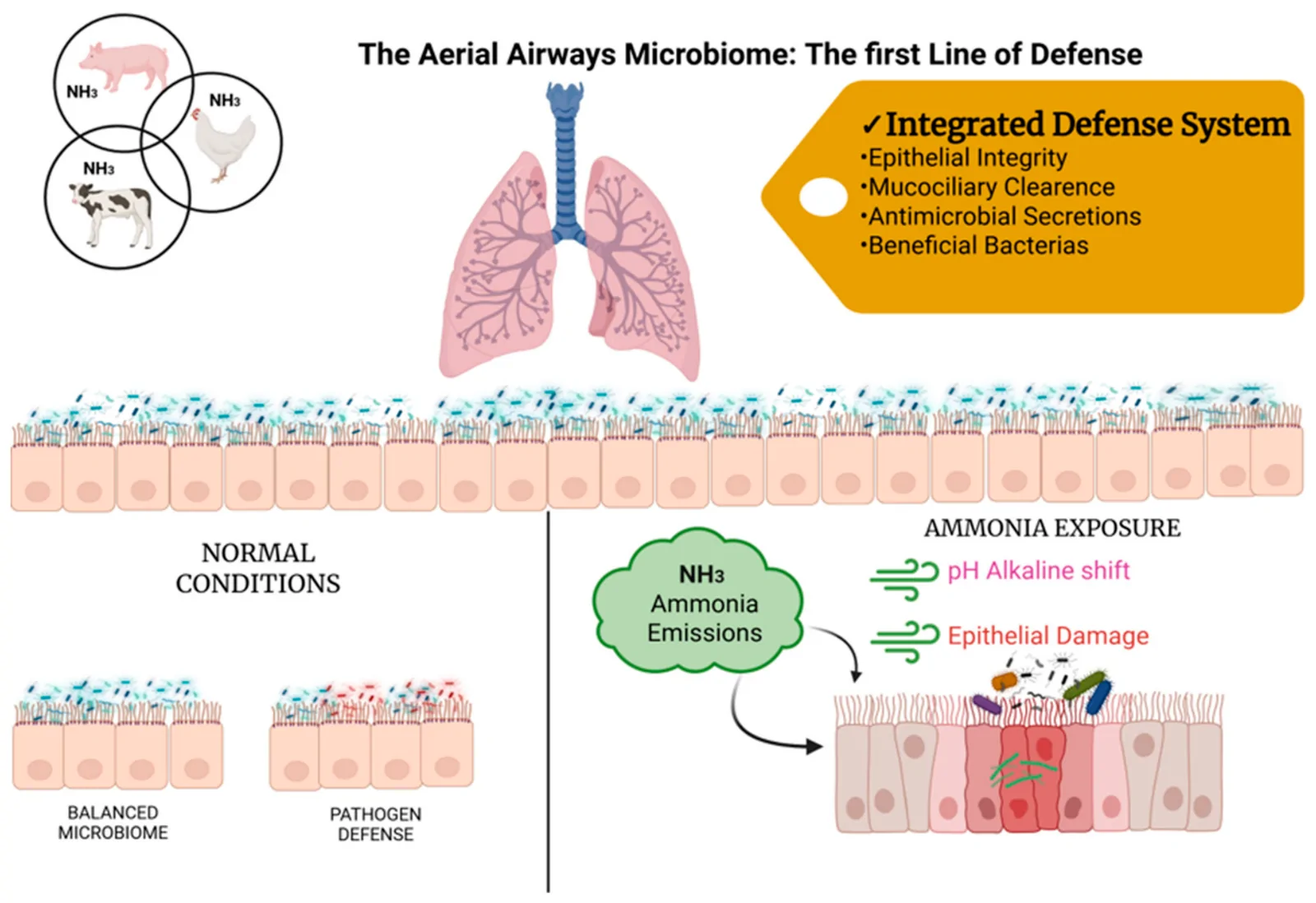
The impact of NH_3_ emissions on the Aerial Airways Microbiome. Figure created by the authors.

The available evidence does not support a single, universally applicable mechanism of ammonia-induced microbiome disruption across targeted livestock species. Although epithelial injury, inflammatory activation, oxidative stress, and microbial community alterations have been reported in broilers and pigs exposed to NH_3_ (Figure 2) [14,21,22,23,24], the strength and nature of this evidence differ substantially between species (Table 1). In cattle, the available information is derived mainly from studies of barn air quality and from broader respiratory microbiome research rather than from direct NH_3_–microbiome experiments [25,26,27,28,29,30,31]. Differences in respiratory anatomy, gastrointestinal physiology, housing systems, exposure patterns, age, diet, and baseline microbiota prevent direct extrapolation of findings from one species to another. Therefore, the responses identified in the current literature should be interpreted separately for each species rather than integrated into a unified livestock-wide mechanistic model.

In avian species, particularly broiler chickens, the evidence is comparatively extensive and is derived mainly from controlled exposure experiments. Most controlled avian studies have reported alterations in intestinal, tracheal, or pulmonary microbial communities following NH_3_ exposure [14,21,23,24,33,35,36,37,38]. However, one short-term exposure study detected increased pulmonary inflammation without significant alterations in lung microbial composition, indicating that inflammatory and microbial responses do not necessarily occur simultaneously [37]. Reported changes include reduced microbial diversity, increased relative abundance of *Proteobacteria* and potentially opportunistic genera such as *Escherichia–Shigella* and *Streptococcus*, and decreased abundance of taxa commonly associated with intestinal homeostasis, including *Lactobacillus*, *Butyricicoccus*, and members of the *Lachnospiraceae* and *Ruminococcaceae* families [14,21,23,24,33,35,36,37,38]. These microbial alterations have frequently occurred together with increased IL-1β, TNF-α, TLR4/MyD88/NF-κB signalling, epithelial injury, oxidative stress, and impaired growth or feed efficiency. Nevertheless, even within avian studies, responses have varied according to the sampled anatomical site, NH_3_ concentration, exposure duration, age of the birds, and analytical method. Moreover, a substantial proportion of the avian evidence originates from a limited number of research groups using similar broiler strains and exposure models [14,21,23,24,33,35,36,37,38]. Consequently, the avian evidence supports an association between NH_3_ exposure, microbiome disruption, inflammation, and impaired performance, but does not establish that an identical causal pathway operates in all poultry categories or under commercial conditions.

In pigs, the available evidence is more limited and heterogeneous. Controlled studies have shown that aerial NH_3_ exposure can alter jejunal or hindgut microbial communities and microbiota-derived metabolites, while also affecting inflammatory markers, lipid metabolism, tissue integrity, and production performance. Reported microbial responses include changes in hindgut community structure, bile acid metabolism, short-chain fatty acid profiles, and bacterial taxa associated with intestinal metabolic functions [33]. Respiratory and systemic effects, including nasal mucosal injury, ciliary dysfunction, pulmonary oxidative stress, inflammatory activation, chronic tracheitis, and interstitial pneumonia, have also been described following NH_3_ exposure. However, these respiratory studies did not consistently evaluate microbial communities and therefore cannot independently demonstrate that the observed lesions were mediated by microbiome alterations [39,40,41,42]. Thus, the porcine evidence suggests that NH_3_ can affect microbial, metabolic, inflammatory, and productive responses, but the direction and biological importance of these effects remain insufficiently characterized.

The evidence for cattle is markedly different. Although the bovine respiratory microbiome has been extensively studied in relation to bovine respiratory disease, transport, feedlot entry, antimicrobial exposure, diet, and other management stressors, direct studies investigating the effects of controlled aerial NH_3_ exposure on bovine microbial communities are currently extremely scarce or absent [28,29,30,43]. Available cattle studies have mainly evaluated NH_3_ concentrations as one component of overall barn air quality and examined associations with respiratory signs, lung consolidation, airway inflammation, infection, calf morbidity, or mortality [25,26,27]. For example, prolonged exposure to barn concentrations above approximately 4 ppm was associated with mild ultrasonographic lung lesions in group-housed calves, whereas concentrations below 6 ppm were associated with a reduced risk of respiratory disease in dairy calves [25,26]. However, these observational associations do not demonstrate that the respiratory effects were caused by microbiome alterations or by NH_3_ independently of other barn contaminants.

Ammonia exposure in commercial cattle housing is closely correlated with ventilation, humidity, temperature, bedding quality, stocking density, airborne particulate matter, endotoxins, and microbial aerosol concentrations [25,26,27]. These interacting environmental factors make it difficult to isolate the independent contribution of NH_3_. Furthermore, the available knowledge regarding bovine respiratory microbial dysbiosis is predominantly derived from studies of bovine respiratory disease and feedlot-associated stress rather than from experimental NH_3_ exposure [28,29,30,31,43].

Accordingly, all cattle-related microbiome interpretations presented in this review should be regarded as limited, hypothesis-generating speculations rather than established conclusions. It is biologically plausible that NH_3_-induced epithelial irritation or impaired mucociliary clearance could modify microbial colonization and promote the expansion of respiratory pathobionts such as *Mannheimia*, *Pasteurella*, or *Histophilus*. However, this proposed sequence has not been directly demonstrated in cattle exposed to defined NH_3_ concentrations. The involvement of these genera in bovine respiratory dysbiosis is well documented [28,29,30,31,43], but their response specifically to aerial NH_3_ exposure remains unknown. Evidence that transport, viral infection, commingling, or other stressors alter the bovine respiratory microbiome cannot be used as direct evidence of an NH_3_-specific microbiome effect [28,29,30,31,43]. Similarly, findings obtained in broilers and pigs should not be extrapolated to cattle without species-specific experimental validation.

### 3.2. Evidence Levels and Directionality of Gut–Lung Communication

The studies included in this review were classified according to their level of mechanistic evidence as either correlational analyses, in which microbial alterations were associated with inflammatory, barrier, metabolic, or production outcomes without direct microbiota manipulation; or causal validation experiments, in which the microbiota was experimentally depleted, transferred, or specifically manipulated to determine whether modification of the microbial community altered the host phenotype.

Most of the included NH_3_–microbiome studies were classified as correlational analyses [14,21,23,24,33,35]. These studies identified associations among NH_3_ exposure, changes in microbial diversity or taxonomic composition, inflammatory biomarkers, epithelial injury, and production performance but did not manipulate the microbiota independently of NH_3_ exposure. For example, associations between increased *Escherichia–Shigella* abundance and impaired growth performance or between altered respiratory taxa and increased IL-1β expression suggest possible microbiota–host interactions but do not demonstrate that the microbial changes caused inflammation or production losses [14,21,23]. In these studies, dysbiosis may have represented a cause, a consequence, or an adaptive response to epithelial injury, inflammation, reduced feed intake, or altered host metabolism. Therefore, these findings should be described as associations rather than confirmed mechanistic pathways.

Three included studies provided interventional evidence of different strengths. Two NH_3_-exposure studies in broilers provided comparatively stronger causal evidence through antibiotic-mediated microbiota depletion and microbiota transplantation [24,32], whereas one porcine study provided partial causal evidence through targeted probiotic intervention [31].

In the first broiler experiment, intestinal microbiota collected from NH_3_-exposed donors were transferred to recipient broilers, resulting in intestinal injury accompanied by increased TLR4 and TNF-α expression. Conversely, antibiotic-mediated microbiota depletion attenuated NH_3_-associated intestinal injury and reduced the expression of TLR4 and TNF-α [24]. These findings support a contributory role of the intestinal microbiota in NH_3_-induced intestinal inflammation through a pathway involving TLR4/TNF-α signalling. However, because germ-free animals and targeted microbial reconstitution were not used, the specific causal taxa, microbial products, and host mediators responsible for this effect remain unidentified.

In the subsequent broiler study [24], antibiotic depletion of the intestinal microbiota reduced the severity of NH_3_-induced tracheal injury, whereas transplantation of microbiota from NH_3_-exposed donors induced tracheal injury and activated TLR4/MyD88/NF-κB signalling even in recipients that were not directly exposed to NH_3_. This experiment provides direct support for an intestinal microbiota contribution to NH_3_-associated tracheal injury in broilers. Nevertheless, the evidence remains limited to a single avian experimental model. Antibiotic treatment may exert microbiota-independent effects, while conventional microbiota transplantation transfers complex microbial communities together with metabolites, bacteriophages, and other fecal components. Consequently, the participation of particular taxa, metabolites, or immune mediators in this pathway remains to be established.

A porcine study provided partial interventional evidence through targeted probiotic supplementation in NH_3_-exposed weaned piglets [34]. Oral administration of *Lactobacillus salivarius* or *Lactobacillus reuteri* altered both ileal and pulmonary microbial communities and was accompanied by changes in pulmonary immune markers and improved production outcomes. FISH and qPCR analyses provided additional support for the presence of probiotic-associated bacteria in the respiratory tract. However, because the experiment did not use germ-free animals, microbiota depletion, fecal microbiota transplantation, strain-specific lineage tracing, or recipient animals receiving isolated microbial communities, it does not establish that microbial translocation was solely responsible for the pulmonary effects. This study should therefore be considered partial causal evidence for a probiotic-mediated gut-to-lung effect rather than definitive validation of the complete NH_3_-associated gut–lung pathway.

#### 3.2.1. Experimentally Supported Gut-to-Lung Pathways

Within the specific context of NH_3_ exposure, the experimentally supported direction of communication is predominantly from the intestinal microbiota toward the respiratory tract. The available evidence supports the following restricted sequence in broilers: NH_3_ exposure alters the intestinal microbiota; the altered microbial community contributes to activation of tracheal TLR4/MyD88/NF-κB signalling; and this response is accompanied by increased TNF-α, IL-1β, IL-6, mucin production, epithelial-barrier disruption, and tracheal injury [24]. This pathway is supported by microbiota depletion and transplantation experiments but has not been validated in germ-free broilers, pigs, or cattle. Moreover, the specific microbial taxa or metabolites responsible for initiating this signalling cascade remain unknown.

The porcine probiotic intervention provides additional, but more limited, support for gut-to-lung communication under NH_3_ exposure [24]. The observed alterations in pulmonary microbiota and immune markers following oral probiotic administration are consistent with an intestinally mediated respiratory effect. However, the absence of strain-specific tracing and microbiota-transfer experiments prevents definitive confirmation of the translocation route and of the causal relationship between the detected pulmonary bacteria and the immune response.

Additional livestock studies not specifically involving NH_3_ provide independent support for a biologically functional gut-to-lung axis. A comparison between germ-free and conventional chickens showed that the intestinal microbiota and the microbiota-derived metabolite butyrate influenced pulmonary metabolism and antiviral immunity during avian influenza virus infection [44]. Antibiotic-mediated modification of the intestinal microbiota also reduced PM_2.5_-induced pulmonary inflammation in broilers, apparently by attenuating dendritic-cell activation and T-cell differentiation. Moreover, fecal microbiota transplantation modified pulmonary Th17/Treg-associated responses and reduced LPS-induced lung injury in broilers [45]. A recent targeted reconstitution experiment showed that oral administration of *Bacteroides uniformis*, or supplementation with the associated microbial metabolite propionate, attenuated PM_2.5_-induced pulmonary inflammation and Th1 polarization [46]. These studies strengthen the biological plausibility of microbiota-mediated gut-to-lung communication in poultry. However, because the experimental challenges involved avian influenza virus, LPS, or particulate matter rather than NH_3_, their mechanisms should not be presented as direct evidence of an NH_3_-specific pathway.

Fecal microbiota transplantation experiments in other respiratory disease models also support a possible gut-to-lung contribution. Transfer of microbiota from *Mycoplasma gallisepticum*-infected chickens impaired intestinal barrier function and host defence against secondary *Escherichia coli* infection, whereas *Lactobacillus salivarius* supplementation reduced pulmonary inflammation [47]. In pigs coinfected with porcine reproductive and respiratory syndrome virus and porcine circovirus type 2, fecal microbiota transplantation was associated with reduced morbidity and mortality [48]. Nevertheless, these studies did not evaluate NH_3_ exposure and therefore provide contextual biological support rather than direct validation of ammonia-induced gut–lung crosstalk.

#### 3.2.2. Hypothetical Lung-to-Gut Pathways

The reverse lung-to-gut direction remains considerably less substantiated in NH_3_-exposed livestock. Across the included evidence, NH_3_ exposure has been associated with respiratory epithelial injury and, in separate studies, alterations in intestinal microbial communities [14,21,23,33,35]. However, the coexistence of pulmonary inflammation and gut dysbiosis does not establish that signals originating in the respiratory tract caused the intestinal alterations. Reduced feed intake, systemic stress responses, altered host metabolism, direct absorption of NH_3_, or other exposure-related effects could also explain these observations.

It may be hypothesized that respiratory inflammation promotes intestinal dysbiosis through circulating cytokines, neuroendocrine responses, immune-cell trafficking, or altered epithelial permeability. Similarly, gut-derived lipopolysaccharides, short-chain fatty acids, bile acids, and other microbial metabolites could potentially influence respiratory immunity after entering the systemic circulation. However, these proposed mediators have not been causally validated in NH_3_-exposed livestock using germ-free animals, sterile fecal filtrates, metabolite-blocking experiments, strain-specific tracing, or targeted microbial reconstitution. Therefore, the bidirectional gut–lung axis in the context of NH_3_ exposure should currently be regarded as a partially supported conceptual framework: limited causal evidence exists for the gut-to-respiratory direction in broilers, whereas the respiratory-to-gut direction remains predominantly hypothetical.

No NH_3_–microbiome studies using germ-free animals, microbiota depletion, fecal microbiota transplantation, or controlled microbiota reconstitution were identified in pigs or cattle. One porcine probiotic intervention provided partial evidence of gut-to-lung communication but did not isolate microbiota-mediated causality or definitively establish the route of bacterial translocation [34]. Consequently, NH_3_-associated gut–lung mechanisms in pigs remain incompletely validated, while those proposed for cattle remain entirely speculative and should not be extrapolated from broiler experiments without species-specific validation.

### 3.3. The Role of Precision Livestock Farming (PLF) in Preserving Microbial Equilibrium

**Figure 3:**
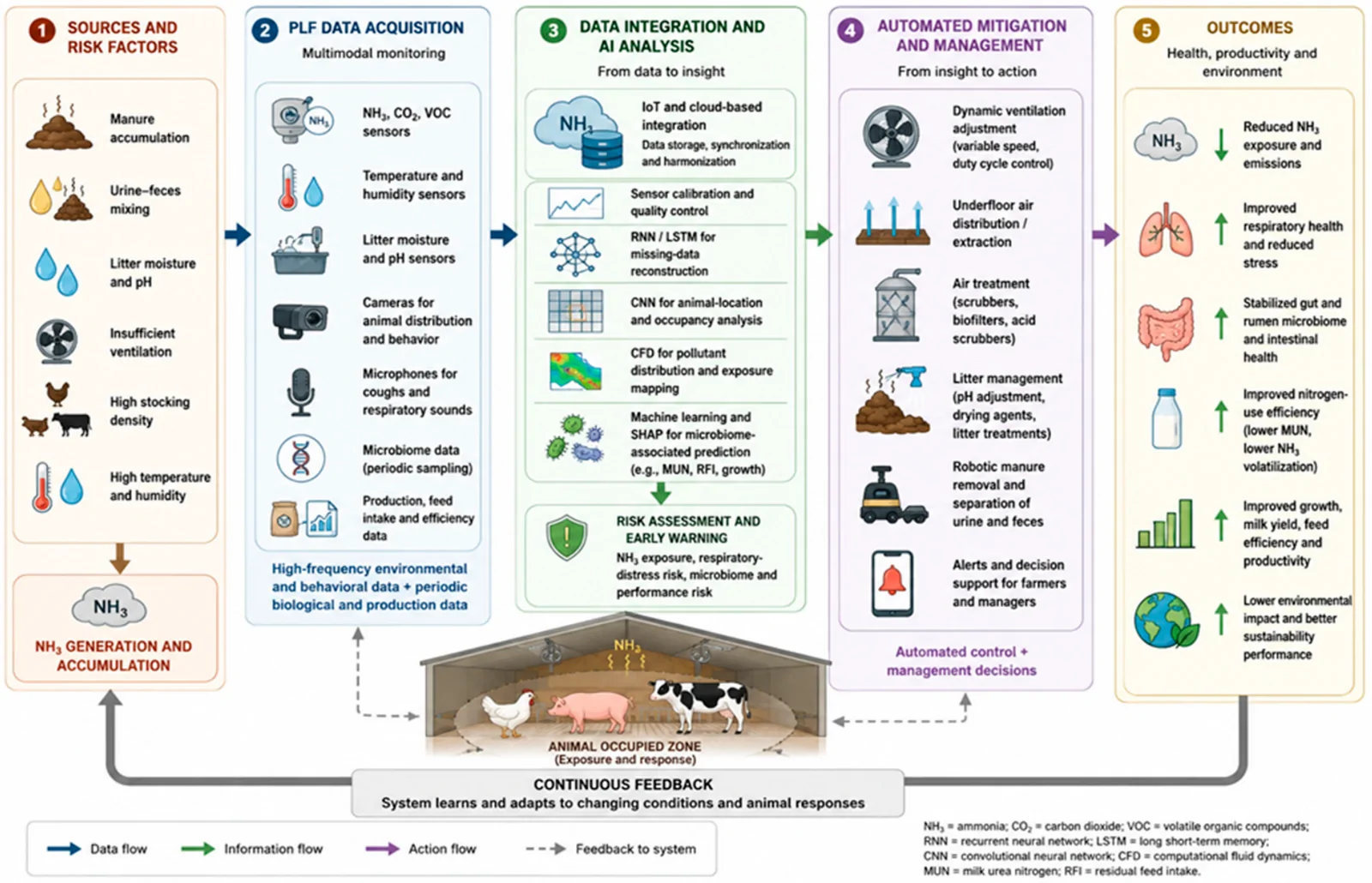
PLF framework for NH_3_ monitoring, risk assessment, and automated reduction in livestock production systems. Figure created by the authors.

The integration of PLF represents a digital transformation in animal husbandry, shifting management from traditional group-based observation to high-frequency, individual-level monitoring [49,50]. In the context of high NH_3_ environments, PLF acts as a critical intervention tool to mitigate the adverse effects of gaseous pollutants on the respiratory and gastrointestinal health of pigs, poultry, and cows [51,52,53]. To effectively protect mucosal integrity, PLF must be conceptualized not merely as a network of sensors, but as a biological closed-loop system encompassing three core stages: real-time ammonia monitoring, microbiome biomarker early warning, and intelligent ventilation intervention (Figure 3). The main PLF technologies currently deployed for NH_3_ monitoring, environmental prediction, and automated mitigation are summarized in Table 2. To accurately assess the industrial viability of these systems, this table outlines the practical bottlenecks frequently encountered in large-scale commercial farms, including sensor drift, dust interference, and the generalization limits of predictive models. Furthermore, Table 2 incorporates a core evaluation metric detailing the suitability of each technology to serve as an early warning. An emerging omics–PLF framework could further integrate continuous environmental monitoring with longitudinal animal phenotypes and molecular biomarkers, thereby linking NH_3_ exposure more directly with biological responses. Real-time measurements of NH_3_, temperature, humidity, animal behaviour, feed intake, and performance could be combined with microbiome profiles, inflammatory and epithelial-barrier markers, and metabolomic or other multi-omics data. Predictive modelling of these integrated datasets may enable the identification of biological changes preceding overt clinical or production impairment and support early-warning systems capable of triggering timely environmental or management interventions. However, NH_3_-specific omics–PLF models remain largely conceptual, and their practical implementation will require standardized longitudinal studies integrating environmental, phenotypic, microbiome, and molecular data. Importantly, continuous environmental monitoring should be temporally aligned with repeated biological measurements rather than interpreted independently. Linking cumulative NH_3_ exposure and transient concentration peaks with longitudinal microbiome profiles, inflammatory and epithelial-barrier biomarkers, metabolomic signatures, and animal performance could help determine whether molecular or microbial alterations precede overt tissue injury. Such synchronized environmental–biological datasets may also improve the identification of biologically relevant exposure thresholds and strengthen predictive early-warning models.

### 3.4. Real-Time Ammonia Monitoring

Accurate, continuous quantification of ammonia is a prerequisite for understanding its role in shifting the host microbiome and inducing systemic inflammation [53,54]. Rather than relying on periodic manual sampling, modern PLF utilizes continuous sensor networks to track spatial and temporal NH_3_ H3 fluctuations. Capturing these real-time environmental dynamics is critical, as transient spikes in NH_3_ concentrations can initiate mucosal irritation, ciliary dysfunction, and early-stage dysbiosis long before structural tissue damage becomes clinically apparent [55,56].

### 3.5. Microbiome Biomarker Early Warning

The core scientific value of integrating PLF into respiratory health management lies in translating environmental monitoring data into predictive biological warnings. A burgeoning frontier in PLF is the use of machine learning algorithms to cross-reference continuous NH_3_ exposure data with longitudinal microbial signatures, predicting the onset of gastrointestinal and respiratory dysbiosis before overt clinical symptoms manifest.

Recent experimental models integrating artificial intelligence with respiratory microbiota have successfully demonstrated that digital monitoring can predict disease states and subclinical inflammation prior to severe tissue damage. In cattle, consensus machine learning approaches utilizing Random Forest algorithms have been deployed to identify differentially abundant microbial variants, serving as predictive biomarkers for bovine respiratory disease [57]. Similarly, in poultry, interpretable machine learning frameworks have successfully extracted stable microbial signatures from the nasal cavity, maintaining high predictive accuracy even under the severe biological stress of highly pathogenic viral infections [58]. To directly link these microbial shifts to physical injury, advanced AI models have been used to integrate respiratory microbiome profiles with host transcriptomics, successfully correlating the expansion of opportunistic pathobionts with the upregulation of host genes responsible for mucosal inflammation and tissue damage [59]. Together, these AI-driven approaches establish a direct predictive link between environmental monitoring, microbial equilibrium, and the prevention of respiratory lesions.

Furthermore, automated acoustic monitoring provides a complementary early-warning mechanism. By detecting subclinical respiratory distress sounds, such as “rales” or coughing triggered by initial mucosal irritation, these systems signal impending airway dysbiosis, allowing for intervention before a primary environmental insult escalates into a secondary polymicrobial infection [60,61].

### 3.6. Intelligent Ventilation Intervention

The final step in the PLF closed-loop system is automated environmental mitigation. Once predictive models detect conditions highly conducive to microbial stress, PLF systems trigger intelligent ventilation protocols to restore Indoor Air Quality (IAQ) and protect mucosal barriers [62,63].

Unlike basic thermostat-linked fans, intelligent ventilation dynamically adjusts airflow based on real-time animal positioning and localized pollutant accumulation, effectively diluting NH_3_ in the specific zones occupied by the animals [62,64]. In broiler production, this is exemplified by the Combined Stress Index (CSI), which integrates sensor data for litter temperature, moisture, and pH [60,65]. Because the CSI correlates strongly with atmospheric NH_3_, it acts as a dynamic threshold indicator that automatically triggers increased ventilation rates or targeted litter treatments when conditions favor microbial urease activity [66,67]. By directly linking digital monitoring strategies to automated environmental controls, PLF provides a vital mechanism for preserving microbial equilibrium and preventing the inflammatory cascades associated with high-ammonia stress [63,68].

**Table 2:** PLF technologies for NH_3_ monitoring, predictive modelling, and automated mitigation in livestock production systems, highlighting practical commercial limitations and suitability for early warning of microbiome dysbiosis.

PLF Component	Data Collected	Analytical Approach	Main Output/Intervention	Livestock Application	Main Limitations	Suitability for Microbiome Early Warning	References
MOS gas sensors and virtual sensor arrays	$NH_{3}$, VOCs, temperature, humidity	Temperature-cycled operation and machine-learning classification	Continuous $NH_{3}$ detection and discrimination from interfering gases	Pig, poultry, and cattle housing	Cross-sensitivity to other farm gases, frequent sensor drift requiring strict recalibration, and biofouling/sensor blinding from airborne dust.	Moderate to High. Can be paired with predictive AI models to track chronic $NH_{3}$ exposure thresholds known to precede respiratory dysbiosis.	[69,70]
Electrochemical sensor-based portable monitoring	$NH_{3}$ concentration, airflow and ventilation rate	Redundant sensing and emission-rate calculations	Estimation of $NH_{3}$ exposure and emission rates	Mechanically ventilated pig and poultry buildings	High sensitivity to barn humidity and severe dust interference leading to rapid membrane degradation and reduced lifespan in commercial settings.	Moderate. Provides accurate spatial exposure data but requires integration with intermittent biological sampling to correlate with mucosal injury.	[55,56]
Environmental sensor networks with missing-data reconstruction	$NH_{3}$, $CO_{2}$, temperature and humidity	RNN and LSTM-based time-series modelling	Data imputation, trend prediction and early-warning alerts	Intensive livestock buildings	Poor generalization of prediction models across different commercial farms; highly dependent on continuous, high-quality training data without prolonged sensor blackouts.	High. Excellent for predicting prolonged environmental stress events and identifying exact timeframes for prophylactic microbial/nutritional interventions.	[64,71]
CFD-integrated monitoring	Airflow, pollutant dispersion and animal-zone exposure	Computational fluid dynamics	Identification of high-exposure zones and ventilation redesign	Poultry houses, pig compartments, and cattle buildings	Extreme computational complexity; static models often fail to account for dynamic farm changes like animal growth, shifting manure piles, or equipment blockages.	Low to Moderate. Better suited for facility redesign than real-time biological warning, though useful for identifying “high-risk” pens/zones for targeted swabbing.	[53,72]
Computer vision-based ventilation control	Animal location and spatial distribution	CNN-based image analysis	Selection of ventilation modes according to occupied zones	Pig and poultry housing	Lens occlusion from farm dust/insects, highly variable lighting conditions, and poor generalization of behavioral algorithms across different breeds or stocking densities.	Low. Primarily tracks behavioral avoidance and thermal comfort rather than predicting subclinical microbial shifts.	[62]
Reinforcement-learning ventilation control	Temperature, humidity, $NH_{3}$, $CO_{2}$, and fan status	DDQN and related reinforcement-learning approaches	Dynamic regulation of indoor air quality and energy consumption	Pig houses and mechanically ventilated facilities	Lack of commercial validation at scale; algorithms may prioritize energy savings over optimal air quality if safety constraints are poorly tuned.	High. If trained with microbiome stress thresholds, it can automatically “close the loop” by increasing ventilation before dysbiosis thresholds are breached.	[73,74]
Microbiome-based predictive modelling	Rumen or intestinal microbiome composition, production and nitrogen-use traits	Metagenomics, ensemble learning, SHAP and transfer learning	Prediction of feed efficiency, nitrogen utilization and emission-related traits	Dairy cattle and pigs	Prohibitive sequencing costs for routine farm use, delays in data turnaround, biological confounding factors (diet, genetics), and poor cross-farm generalization.	Very High (Direct). Allows for the direct detection of microbial biomarkers (e.g., pathobiont expansion), enabling immediate targeted interventions.	[75,76,77]

### 3.7. Environmental Controls and Ventilation Optimization

Environmental management remains a cornerstone of respiratory disease prevention and microbiome preservation in intensive livestock production systems [78]. Among airborne environmental stressors, ${\mathrm{NH}}_{3}$ is particularly detrimental due to its direct cytotoxic effects on the respiratory epithelium and its capacity to disrupt host-associated microbial equilibrium [79]. Consequently, environmental abatement strategies aimed at reducing indoor ${\mathrm{NH}}_{3}$ accumulation are critical for maintaining airway health and can be evaluated based on their quantitative reduction efficacy, protective capacity for mucosal barriers, retention of microbial diversity, and economic viability [80]. A comprehensive quantitative summary of these environmental control approaches is provided in Table 3.

#### 3.7.1. Precision Ventilation and Airflow Optimization

Ventilation optimization plays a central role in controlling indoor air quality and regulating thermal microenvironments in livestock housing [81]. Adequate mechanical ventilation facilitates the rapid removal of noxious gases, airborne particulates, and excess moisture, thereby reducing overall microbial load and limiting host exposure to respiratory irritants [82]. In poultry housing systems, insufficient ventilation has been consistently associated with elevated ${\mathrm{NH}}_{3}$ concentrations, increased incidence of tracheal and pulmonary lesions, and impaired production performance [3,83].

Computational fluid dynamics (CFD) modeling and field evaluations demonstrate that increasing indoor ventilation rates from $3.3{\;m}^{3}/s$ (0.1 m/s inlet velocity) to $4.2{\;m}^{3}/s$ (1.0 m/s inlet velocity) dramatically decreases average indoor ${\mathrm{NH}}_{3}$ concentrations from 72.87 ppm down to 12.3 ppm [81]. Furthermore, implementing transient duty-cycle variable ventilation strategies allows producers to maintain target ${\mathrm{NH}}_{3}$ levels (15–20 ppm) while achieving 11.8% to 23.6% reductions in daily ventilation energy consumption [3,83].

For total exhaust air treatment, advanced mechanical ventilation systems can be integrated with acid air scrubbers [84]. Acid scrubbers utilizing recirculating acidic water (pH 1.0–5.5) convert gaseous ${\mathrm{NH}}_{3}$ into non-volatile ammonium (${\mathrm{NH}}_{4}^{+}$), capturing 75% to 95% of total exhaust ${\mathrm{NH}}_{3}$ emissions [80,84]. Partial pit ventilation systems—which extract 10% to 20% of the ventilation volume directly from the headspace above slurry pits—capture 65% to 75% of total indoor ${\mathrm{NH}}_{3}$ emissions before the gas reaches the animal-occupied zone [84]. Although air scrubbers require substantial initial capital expenditure (CAPEX) and ongoing operational energy (OPEX), the long-term economic return on investment (ROI) is supported by improved feed conversion ratios, reduced mortality, and the potential recovery of nitrogen mineral concentrates (RENURE) as sustainable fertilizers [80,84].

#### 3.7.2. Chemical and Biological Litter/Manure Acidification

Management practices such as litter and slurry acidification represent highly effective chemical and biological interventions to suppress ${\mathrm{NH}}_{3}$ volatilization [36,83]. Acidifying manure or bedding shifts the chemical equilibrium toward non-volatile ammonium (${\mathrm{NH}}_{4}^{+}$) while inhibiting the enzymatic activity of microbial urease and uricase [4,26]. Lowering litter or slurry pH below 6.0 (ideally target pH 5.5) suppresses the growth of uricolytic and ureolytic bacteria, preventing acute mucosal irritation, ciliary desquamation, and respiratory lesions in poultry and swine [3,85].

Chemical Acidification: In-barn chemical slurry acidification using inorganic or organic acids (e.g., sulfuric, acetic, or lactic acid) achieves an in-house ${\mathrm{NH}}_{3}$ emission reduction of 56% to 85%, and an overall chain reduction exceeding 90% [84,85]. In poultry operations, surface-applied acidifiers (e.g., sodium bisulfate) lower litter pH, delaying the onset of atmospheric ${\mathrm{NH}}_{3}$ spikes during critical early brooding weeks [3,83].

Biological Acidification (LAB SynCom): In situ biological acidification using a synthetic lactic acid bacteria community (LAB SynCom, comprising *Enterococcus durans*, *Lactobacillus casei*, *Lactobacillus paracasei*, and *Lactobacillus plantarum*) achieves a total cumulative ${\mathrm{NH}}_{3}$ reduction efficiency of 95.5% over 12 days of manure storage [85]. This biological intervention maintains manure pH between 5.58 and 6.05 through sustained lactic acid generation, directly suppressing 22 key ureolytic bacterial genera (including *Lysinibacillus*, *Alcaligenes*, *Proteiniphilum*, *Comamonas*, and *Acinetobacter*) and preserving nitrogen content within the slurry [85].

#### 3.7.3. Automated Manure Removal, Storage Covers, and Land Application

In swine and cattle facilities, automated manure management systems target the primary biochemical driver of ${\mathrm{NH}}_{3}$ formation by preventing the prolonged mixing of urinary urea and fecal urease [84,86]. High-frequency automated robotic scrapers or urine-feces source-segregation systems reduce in-house ${\mathrm{NH}}_{3}$ emission rates by 30% to 50%. Rapidly transferring fresh slurry from animal housing to closed external storage facilities achieves an overall manure-chain ${\mathrm{NH}}_{3}$ reduction of 46% [84].

At the storage and field application stages, additional abatement measures prevent downstream losses [80]. Storage Cover Regimens: Fitting external slurry tanks with solid roofs, lids, or tensioned tents achieves an 80% reduction in ${\mathrm{NH}}_{3}$ emissions. Flexible floating plastic sheets reduce emissions by 60%, natural crust layers (>7% dry matter) achieve a 35–50% reduction, and floating light expanded clay aggregate (LECA) or oil layers achieve a 40% reduction. Field Application Techniques: Applying slurry using trailing-hose band spreaders yields a 10–50% (30% average) reduction, trailing-shoe applicators yield a 40–70% reduction, open-slot shallow injection achieves a 60% reduction, and closed-slot deep injection achieves an 80–90% reduction. Immediate soil incorporation of broadcasted manure via ploughing or disc harrows within 0–4 h of spreading achieves an 80% to 90% reduction in ${\mathrm{NH}}_{3}$ volatilization [80].

#### 3.7.4. Upstream Interventions as “Microbiome Rescue

Collectively, these environmental interventions not only protect epithelial integrity and mucociliary clearance but also indirectly support microbial homeostasis by minimizing ecological disturbances within the airway microbiome [87]. By preventing high airborne ${\mathrm{NH}}_{3}$ exposure, environmental control strategies act upstream as essential components of “microbiome rescue”, preserving commensal diversity and preventing the shift toward pathobiont-dominated dysbiosis rather than attempting to correct mucosal damage after its clinical onset [85,87].

**Table 3:** Quantitative comparison of environmental control strategies on ammonia reduction, respiratory mucosal health, microbial retention, and economic viability.

Environmental Control Strategy	Indoor NH_3_ Decreasing Range	Alleviation of Mucosal Lesions	Retention Level of Microbial Diversity	Cost and Economic Benefit Profile	References
Ventilation Retrofitting (Precision Airflow & Scrubbers)	75% to 90% removal using acid air scrubbers. Modulating inlet velocities from 0.1 m/s to 1.0 m/s decreases average indoor NH_3_ from 72.87 ppm to 12.3 ppm (approx. 83% reduction).	Prevents prolonged exposure to high NH_3_ levels (>25 ppm) that cause keratoconjunctivitis, tracheal irritation, and loss of cilia in poultry.	Limits environmental stress, preventing ammonia-induced dysbiosis (such as the overgrowth of *Streptococcus* and depletion of *Lactobacillus*).	Requires considerable initial investment and energy costs. However, variable ventilation duty cycles yield 11.8% to 23.6% in daily energy savings, and captured nitrogen can be economically valorized as “Renure” (mineral fertilizer).	[3,23,81,84]
Litter & Manure Acidification (Chemical & Biological)	56% to 85% in-barn reduction via chemical acidification. Biological acidification using a synthetic lactic acid bacteria community (LAB SynCom) achieves a 95.5% total reduction over 12 days.	Lowers litter pH, delaying the onset of atmospheric NH_3_ spikes and preventing respiratory lesions, airsacculitis, and secondary infections.	Biological acidification reshapes the manure microbiome, stably colonizing it with *Lactobacillus* and *Enterococcus* while specifically inhibiting 22 ureolytic bacterial genera.	Low-dose chemical acidification is an economically viable strategy for emission mitigation. Biological LAB treatments offer a cost-effective, environmentally friendly alternative to corrosive sulfuric acids.	[3,83,84,85]
Automated Manure Removal & Separation	46% reduction across the manure chain via rapid removal and central digestion. Up to 75% reduction when combined with active feces-urine segregation (e.g., Sphere system).	Decreases chronic ambient exposure to noxious gases, protecting epithelial tight junctions and reducing overall respiratory distress.	Minimizes systemic stress, thereby indirectly preserving the homeostatic balance of the gut-lung microbial axis.	Very high capital expenditure (CAPEX) for robotic scrapers and digesters; however, it provides strong long-term returns through reduced labor, improved animal welfare, and biogas (renewable natural gas) revenue.	[84,87]

## 4. Discussion

The findings of this systematic review indicate that environmental NH_3_ exposure can alter respiratory and gastrointestinal microbial communities in livestock, although the strength and nature of the evidence differ considerably among species. The most consistent evidence derives from controlled studies in broiler chickens, in which NH_3_ exposure was associated with changes in microbial diversity and community composition in the intestine, trachea, and lungs [14,33,35,36,37,38]. These alterations frequently involved increased abundance of potentially opportunistic taxa, including *Escherichia–Shigella* and *Streptococcus*, together with reductions in taxa commonly associated with microbial homeostasis, such as *Lactobacillus*, *Butyricicoccus*, and members of *Lachnospiraceae* and *Ruminococcaceae*. Microbial alterations were also frequently accompanied by epithelial injury, oxidative stress, inflammatory activation, and impaired production performance, including activation of TLR4/MyD88/NF-κB-related pathways. However, the coexistence of dysbiosis and inflammation does not necessarily establish causality, as microbial changes may represent a cause, consequence, or adaptive response to epithelial injury, inflammation, altered feed intake, or metabolic disturbances. This is supported by a short-term exposure study in which pulmonary inflammation occurred without significant changes in lung microbial composition [37], suggesting that direct NH_3_-induced epithelial and inflammatory effects may precede detectable microbial restructuring. These findings provide an important link across microbial, molecular, and phenotypic levels. At the microbial level, NH_3_ exposure altered the intestinal microbiota; at the molecular level, these changes were associated with activation of TLR4/MyD88/NF-κB and increased inflammatory mediators; and at the phenotypic level, these responses coincided with epithelial-barrier disruption, mucus hypersecretion, apoptosis, and tracheal injury. The attenuation of these effects following microbiota depletion and their reproduction after microbiota transplantation strengthen the interpretation that the altered intestinal microbiota contributes functionally to the respiratory phenotype. However, the specific taxa, metabolites, or microbial products responsible for initiating this signaling cascade remain unresolved.

Importantly, these taxonomic alterations should be distinguished from functional microbiome effects. Changes in the relative abundance of taxa such as *Escherichia–Shigella* or *Streptococcus* are primarily descriptive and do not, by themselves, demonstrate altered microbial activity or causality. Stronger mechanistic evidence is provided when microbial shifts coincide with functional host responses, including activation of TLR4/MyD88/NF-κB and TLR4/TNF-α signalling, altered inflammatory cytokine expression, epithelial-barrier disruption, or microbiota-associated metabolic changes. Nevertheless, the specific microbial metabolites or molecular products linking individual taxa to these host responses remain largely unidentified in the available studies. Thus, current evidence supports an association between taxonomic dysbiosis and functional host alterations, but only a limited number of studies establish a direct microbiota-mediated mechanism.

Compared with poultry, evidence in pigs is more limited. Controlled studies nevertheless suggest that aerial NH_3_ exposure can modify gastrointestinal microbial communities and microbiota-associated metabolic profiles while affecting intestinal integrity, inflammatory responses, metabolism, and productive performance [88]. Respiratory consequences, including nasal mucosal injury, impaired ciliary function, pulmonary oxidative stress, chronic tracheitis, and interstitial pneumonia, have also been reported [39,40,41,42]. Nevertheless, respiratory injury should not be considered synonymous with respiratory dysbiosis because microbial communities were not consistently characterized in these studies. A probiotic intervention in NH_3_-exposed piglets provided additional evidence for communication between intestinal and pulmonary microbial compartments, although the absence of microbiota depletion, transplantation, or strain-specific tracing prevents definitive attribution of the respiratory effects to gut-derived microbial changes [34].

Evidence in cattle is considerably weaker. Although the bovine respiratory microbiome has been extensively investigated in relation to respiratory disease, transportation, antimicrobial exposure, diet, and other environmental stressors, direct controlled studies evaluating aerial NH_3_-induced alterations of bovine respiratory or gastrointestinal microbiota remain extremely scarce [28,29,30,31,43]. Associations between barn NH_3_ concentrations and respiratory outcomes support the importance of air quality [25,26,27], but they do not demonstrate microbiome-mediated effects. Interpretation is further complicated by the interaction of NH_3_ with ventilation, humidity, temperature, bedding conditions, particulate matter, endotoxins, and airborne microorganisms. Consequently, mechanisms identified in poultry or pigs should not be extrapolated directly to cattle without species-specific experimental validation.

An important finding emerging from the included studies concerns the potential involvement of the gut–lung axis in NH_3_-associated respiratory injury. While most studies remain correlational [14,21,24,33,35], microbiota depletion and transplantation experiments in broilers provide stronger evidence of causality [23,32]. Antibiotic-mediated depletion of intestinal microbiota attenuated NH_3_-associated intestinal and tracheal injury, whereas transplantation of microbiota from NH_3_-exposed donors reproduced inflammatory alterations in recipient birds. Particularly relevant was the observation that microbiota transplantation induced tracheal injury and activation of TLR4/MyD88/NF-κB signalling in recipients without direct NH_3_ exposure [32], supporting a functional contribution of intestinal dysbiosis to respiratory injury. Nevertheless, these experiments cannot completely identify the underlying mechanism because antibiotics may exert microbiota-independent effects and microbiota transplantation transfers microorganisms together with metabolites, bacteriophages, and other biological components. Evidence from livestock models involving other respiratory challenges further supports the biological plausibility of gut–lung communication [44,45,46,47,48,89], but these findings should be considered supportive rather than direct evidence of an NH_3_-specific mechanism. Moreover, the reverse lung-to-gut direction remains poorly demonstrated under NH_3_ exposure. Thus, current evidence supports a partially validated and directionally asymmetric gut–lung relationship, with stronger experimental support for intestinal microbiota contributing to respiratory injury than for respiratory alterations driving intestinal dysbiosis.

Taken together, these findings emphasize that NH_3_-associated microbiome responses should not be generalized across livestock species. Poultry currently provides the strongest experimental evidence, whereas porcine evidence remains comparatively limited and direct bovine evidence is insufficient. Differences in respiratory anatomy, gastrointestinal physiology, diet, housing, exposure conditions, and baseline microbial communities further restrict cross-species extrapolation. Environmental management remains relevant because reducing NH_3_ exposure may prevent the epithelial and ecological disturbances associated with microbial disruption. Improved manure management, litter or slurry acidification, ventilation, and exhaust-air treatment can reduce NH_3_ accumulation [3,80,81,82,84], although these approaches should be regarded as NH_3_-exposure mitigation measures rather than established microbiome-restorative interventions. Similarly, precision livestock farming and continuous environmental monitoring may facilitate earlier identification of harmful exposure conditions [53,60,62,63], but a validated predictive relationship between real-time NH_3_ concentrations and subsequent respiratory or gastrointestinal dysbiosis has not yet been established.

Several limitations should be considered when interpreting these findings. The evidence was markedly unevenly distributed among species: of the eight studies included in the systematic review, five investigated broiler chickens and three investigated pigs, whereas no bovine NH_3_–microbiome study met the eligibility criteria. The additional cattle publications discussed in this section [25,26,27,28,29,30,31,43] were used exclusively to contextualize existing research gaps and should not be interpreted as direct evidence of NH_3_-induced microbiome alterations in cattle. Consequently, the apparent consistency of certain microbial and inflammatory responses may primarily reflect repeated investigation of similar broiler models rather than a conserved biological response across livestock species. The limited number of eligible studies also prevents formal comparisons of species susceptibility and the establishment of reliable species-specific exposure–response thresholds.

Considerable methodological heterogeneity further restricts comparison among studies. Investigations differed in NH_3_ concentrations and measurement units, exposure duration, animal age, housing conditions, diet, sampling sites, sequencing regions, bioinformatic pipelines, and definitions of dysbiosis [14,21,22,23,24,25,26,27,35,37,39,40,41,42,85]. Some experiments performed microbiome sequencing only in the control and highest-exposure groups, whereas others lacked baseline samples, functional microbial measurements, inflammatory biomarkers, or production outcomes [14,21,22,23,24,35,36,37]. Most studies also relied on relative taxonomic abundance determined by 16S rRNA sequencing, which cannot establish microbial activity or distinguish absolute bacterial expansion from depletion of other taxa or mathematical changes in relative abundance. Fungal, viral, archaeal, and strain-level components were rarely investigated.

An additional limitation concerns the interpretation of NH_3_ exposure thresholds. Environmental exposure thresholds should not necessarily be considered equivalent to biological response thresholds, as microbial, epithelial, inflammatory, or metabolic alterations might occur at concentrations below those associated with overt clinical or production effects. Moreover, biological thresholds may vary according to species, age, exposure duration, anatomical compartment, and the endpoint evaluated. Given the heterogeneity of the included studies, the available evidence does not permit identification of a universal NH_3_ concentration at which microbiome dysbiosis or associated host responses are initiated. Future studies should therefore distinguish environmental exposure limits from species- and endpoint-specific biological response thresholds.

The predominance of endpoint or cross-sectional sampling additionally limits temporal and causal interpretation. Associations among NH_3_ exposure, microbial alterations, inflammatory biomarkers, tissue injury, and production losses do not necessarily demonstrate that dysbiosis caused the observed effects. Important research gaps are particularly evident in cattle. Controlled longitudinal studies in calves and adult cattle should evaluate defined and commercially relevant NH_3_ concentrations while controlling for particulate matter, endotoxins, temperature, humidity, ventilation, bedding, diet, antimicrobial exposure, and infectious status. Sampling before, during, and after exposure should encompass respiratory and gastrointestinal compartments, including the nasal cavity, nasopharynx, trachea, bronchoalveolar compartment, rumen, and intestine, and should integrate microbiome analysis with mucosal histopathology, epithelial barrier and inflammatory markers, metabolomics, clinical respiratory assessment, lung ultrasonography, growth, feed efficiency, and milk production. Shotgun metagenomics, metatranscriptomics, metabolomics, and strain-level analyses would also provide greater functional resolution than 16S rRNA sequencing alone. Without such evidence, neither causality nor an NH_3_-specific bovine gut–lung mechanism can be established.

The findings of this review should therefore be interpreted as species-dependent, with the strongest evidence for NH_3_-associated respiratory and gastrointestinal microbiome alterations currently available in broilers, more limited evidence in pigs, and insufficient direct evidence in cattle. Future standardized, controlled, and longitudinal studies are required to distinguish direct NH_3_ toxicity from microbiota-mediated effects and to determine whether a broader ammonia–microbiome–gut–lung mechanism can be established across livestock species.

## 5. Conclusions

This systematic review indicates that environmental NH_3_ exposure is associated with alterations of the respiratory and gastrointestinal microbiome in livestock, although the strength of evidence is markedly species dependent. The most consistent evidence was identified in broiler chickens, where NH_3_ exposure was associated with changes in microbial diversity and composition, disruption of microbial homeostasis, epithelial injury, inflammatory activation, and impaired production-related outcomes. In pigs, available studies similarly support gastrointestinal microbiome alterations and associated inflammatory and metabolic effects, although evidence directly linking intestinal dysbiosis with respiratory microbiome changes remains more limited. Importantly, no bovine NH_3_–microbiome studies fulfilled the eligibility criteria, preventing direct conclusions regarding this relationship in cattle. The available evidence also supports a potential role of the gut–lung axis in NH_3_-associated injury. Microbiota depletion and transplantation experiments in broilers provide the strongest indication that intestinal dysbiosis may contribute to respiratory inflammation, particularly through pathways involving TLR4/MyD88/NF-κB signalling. Nevertheless, most available studies remain associative, and the direction and mechanisms of microbiome–host interactions cannot yet be generalized across target livestock species. Environmental NH_3_ should be considered an important ecological stressor capable of affecting host-associated microbial communities, but current evidence is limited by the small number of eligible studies, methodological heterogeneity, predominance of broiler models, and reliance on endpoint and 16S rRNA-based analyses. Standardized, controlled, and longitudinal studies are needed to establish species-specific exposure–response relationships and distinguish direct NH_3_ toxicity from microbiota-mediated effects. Such evidence will be essential to determine whether a broader ammonia–microbiome–gut–lung mechanism can be established across livestock species.

## Figures and Tables

**Table 1:** Characteristics, microbiome alterations, inflammatory responses, production outcomes, and level of causal evidence in the included studies.

Study	Species	Age/Stage	NH_3_ Level	Duration (Days)	Sampling Site	Core Microbial Alterations	Inflammatory Biomarkers	Production Outcomes	Study Classification and Level of Causal Evidence
[32]	Male Arbor Acres broiler chickens	21 days old at exposure onset; grower phase (21–42 days of age)	Control: <3 ppm; exposure: 35 ppm	21 days	Ileal contents and tracheal tissue for microbiota analysis; tracheal tissue for inflammatory markers	Ileum: decreased Firmicutes; increased Proteobacteria and Bacteroidetes; enrichment of *Escherichia–Shigella*, *Faecalibacterium*, *Streptococcus*, *Ruminococcaceae_UCG-014*, *Rothia*, unclassified *Lachnospiraceae*, *[Ruminococcus]_torques_group* and unclassified *Ruminococcaceae*. Trachea: altered beta diversity and increased *[Ruminococcus]_torques_group*, unclassified *Lachnospiraceae*, *Faecalibacterium*, *Ruminococcaceae_UCG-014* and *Alistipes*.	Increased tracheal TLR4, MyD88, NF-κB, TNF-α, IL-1β, IL-6 and IL-10. Claudin-1 decreased, whereas muc5ac and caspase-3 increased, indicating epithelial-barrier injury, mucus hypersecretion and apoptosis.	Body weight was recorded, but production-performance results were not reported.	Causal validation experiment—antibiotic-mediated microbiota depletion and microbiota transplantation
[33]	Female fattening pigs	125 days old; fattening stage	Control: <5 mg/m^3^; exposure: 88.2–90.4 mg/m^3^, 8 h/day	30 days	Jejunal contents for microbiota; feces for inflammatory biomarkers	No significant changes in alpha diversity, but beta diversity was altered. Increased Clostridia and Alphaproteobacteria; decreased Gammaproteobacteria, Bacteroidia, Fusobacteriia and Actinobacteria. Increased *Pseudomonas*, *Terrisporobacter*, *Brevundimonas* and *Clostridium_sensu_stricto_1*; decreased *Actinobacillus*, *Moraxella*, *Porphyromonas*, *Bergeyella*, *Neisseria* and *Alloprevotella*.	Increased fecal LTF (+178.67%), MPO (+199.44%), MMP-9 (+596.95%) and calprotectin (+261.51%) compared with controls.	Not assessed/reported.	Correlational analysis—no direct microbiota manipulation
[34]	Duroc × Landrace weaned piglets	Nursery/weaning stage; mean initial BW 7.1 kg; chronological age not reported	16–19 ppm; all experimental groups were exposed	28 days	Ileal mucosa from the ileocecal junction and lung tissue	Probiotic supplementation increased microbial diversity in the ileum and lungs. In the ileum, *L. salivarius* increased *Lactobacillus* and *Escherichia–Shigella*, whereas *L. reuteri* increased *Lactobacillus*, *Prevotella* and *Holdemanella*. In the lungs, probiotic supplementation increased *Lactobacillus*, *Actinobacillus*, *Prevotella* and *Prevotellaceae_NK3B31_group*, while untreated piglets had higher *Mycoplasma*, *Escherichia–Shigella* and *Streptococcus*. Increased pulmonary *L. reuteri*, *L. johnsonii* and *L. intestinalis* suggested gut-to-lung translocation.	IL-2, IL-4, IFN-α and TNF-α expression increased in the ileum and lungs following probiotic supplementation; the highest levels were observed in the *L. salivarius* group.	*L. salivarius* and *L. reuteri* increased final BW and average daily gain compared with the untreated control. The lowest feed-to-gain ratio occurred in the *L. salivarius* and aureomycin groups. Probiotics also reduced respiratory/digestive disease incidence and mortality.	Causal validation experiment (partial)—targeted probiotic intervention; gut-to-lung bacterial translocation supported by FISH and qPCR, but not confirmed using germ-free animals or FMT
[24]	Male Arbor Acres broiler chickens	Exposure during 4–6 weeks of age; grower–finisher stage	Control: <3 ppm; exposure: 35 ppm	3 weeks; samples collected at 42 days of age	Ileal contents for microbiota; ileal tissue for histology and inflammatory markers	Increased alpha diversity (Chao and Shannon) and altered beta diversity. Decreased Firmicutes; increased Proteobacteria and Bacteroidetes. Increased *Streptococcus*, *Escherichia–Shigella*, *Faecalibacterium*, *[Ruminococcus]_torques_group*, *Ruminococcaceae_UCG-014*, unclassified *Lachnospiraceae*, *Rothia* and unclassified *Ruminococcaceae*.	Increased TLR4, MyD88, NF-κB, TNF-α and IL-1β; decreased IL-6 and IL-10. Increased caspase-3 and decreased mucin-2 and claudin-1, indicating apoptosis and epithelial-barrier injury.	Decreased average daily feed intake (ADFI) and average daily gain (ADG); increased feed-to-gain ratio (F/G).	Causal validation experiment—antibiotic-mediated microbiota depletion and microbiota transplantation
[35]	Yorkshire × Landrace growing pigs	70 days old; initial BW 20.27 ± 0.36 kg	Control: no added NH_3_; exposure: 35 mg/m^3^	25 days	Cecal and proximal colonic digesta	Increased alpha diversity and altered beta diversity in the cecum and colon. Cecum: increased Bacteroidota and Spirochaetota; decreased Verrucomicrobiota and Cyanobacteria. Increased *Alloprevotella*, *Lachnospira*, *Lactobacillus*, *Butyricicoccus*, *Monoglobus* and *Anaerovibrio*; decreased *Akkermansia*, *Clostridium_sensu_stricto_1*, *Terrisporobacter*, *Ralstonia* and *Peptococcus*. Colon: increased *Lachnospira*, *Fournierella*, *Negativibacillus*, *Monoglobus* and members of *Butyricicoccaceae*; decreased *Terrisporobacter*.	Not assessed/reported	No significant effects on feed intake, body weight or body-weight gain.	Correlational analysis—no direct microbiota manipulation
[23]	Male Arbor Acres broiler chickens	22 days old at exposure start; grower–finisher stage, days 22–42	Control: nominal 0 ppm (<3 ppm measured); treatments: 15 ± 3, 25 ± 3 and 35 ± 3 ppm	21 days	Tracheal tissue/microbiota	Dose-dependent reduction in richness and altered community structure from 15 ppm; *Lactobacillus*; *Faecalibacterium*, *Blautia*, *Ruminococcus torques* group, unclassified *Lachnospiraceae*, *Ruminococcaceae_UCG-014* and *Streptococcus*	Tracheal IL-1β and IL-6 at 15, 25 and 35 ppm; IL-10 at 25 and 35 ppm. Moderate tracheal lesions at 15–25 ppm and severe mucosal deformation, inflammatory-cell infiltration and submucosal edema at 35 ppm	15 ppm: no significant performance effect; 25 and 35 ppm: ADG and final BW; 35 ppm: ADFI and poorer feed conversion (F:G)	Correlational analysis—no direct microbiota manipulation
[14]	Male Arbor Acres broiler chickens	21 days old; grower stage	0 ± 3, 15 ± 3, 25 ± 3 and 35 ± 3 ppm	21 days	Cecal contents; microbiome analyzed only in the 0 and 35 ppm groups at day 21	At 35 ppm: altered beta diversity, but no significant alpha-diversity changes; Proteobacteria and *Escherichia–Shigella*; *Butyricicoccus*, *Parasutterella*, *Lachnospiraceae_UCG-010*, *Ruminococcaceae_UCG-013* and *Ruminococcaceae_UCG-004*	Not assessed in this study	15 ppm: no significant effects. At 25 ppm: F:G after 7 days and BW after 14 days; BWG and BW after 21 days. At 35 ppm: F:G and BWG/BW from day 7; by day 21, ADFI, BWG and BW and markedly F:G	Correlational analysis—no direct microbiota manipulation
[21]	Male Arbor Acres broiler chickens	22 days old at exposure start; grower-finisher stage, days 22–42	0, 15, 25 and 35 ppm	21 days; sampling after 7 and 21 days	Lung tissue/pulmonary microbiota	25 and 35 ppm disturbed pulmonary microbiota; Proteobacteria after 7 and 21 days; unclassified *Ruminococcaceae* significantly altered after 7 days, but not after 21 days	Day 7: IL-1β at 25 and 35 ppm; IL-6 and IL-10 unchanged. Day 21: IL-1β and IL-6 at 35 ppm; IL-10 at 15, 25 and 35 ppm. Additionally, 5-HT at 35 ppm, while norepinephrine was unchanged	Not assessed in this study	Correlational analysis—no direct microbiota manipulation

## Data Availability

No new data were created or analyzed in this study. Data sharing is not applicable to this article.

## Supplementary Materials

The following supporting information can be downloaded at https://www.mdpi.com/article/10.3390/vetsci13090952/s1, Table S1. Database-specific search strategies used for the systematic literature search; Table S2. SYRCLE risk-of-bias assessment of the studies included in the systematic review; Table S3. Base articles; Table S4. Representative examples of articles excluded during title and abstract screening according to the predefined exclusion criteria; Table S5. Representative articles excluded following full-text assessment.

## Abbreviations

The following abbreviations are used in this manuscript:

NH_3_	Ammonia
PRISMA	Preferred Reporting Items for Systematic Reviews and Meta-Analyses
NH_4_^+^	Ammonium
H_2_S	Hydrogen sulfide
OSHA	Occupational Safety and Health Administration
CO_2_	Carbon dioxide
NIOSH	National Institute for Occupational Safety and Health
CBF	Ciliary beat frequency
PM_10_	Concentrations of particulate matter
PRDC	Porcine respiratory disease complex
BRD	Bovine respiratory disease
