# Supplementary material for: Respiratory and Gastrointestinal Microbiome Shifts in Livestock Exposed to High-Ammonia Environments: A Systematic Review
Source: Vet Sci. 2026 Sep 12;13(9):952. doi: 10.3390/vetsci13090952 (PMC13612021; doi:10.3390/vetsci13090952)
Supplement: Supplementary file 1 [file vetsci-13-00952-s001.zip › vetsci-4434675-File S1 PRISMA flow diagram.pdf]

File S1

PRISMA flow diagram for new systematic reviews which included searches of databases only (07.06)

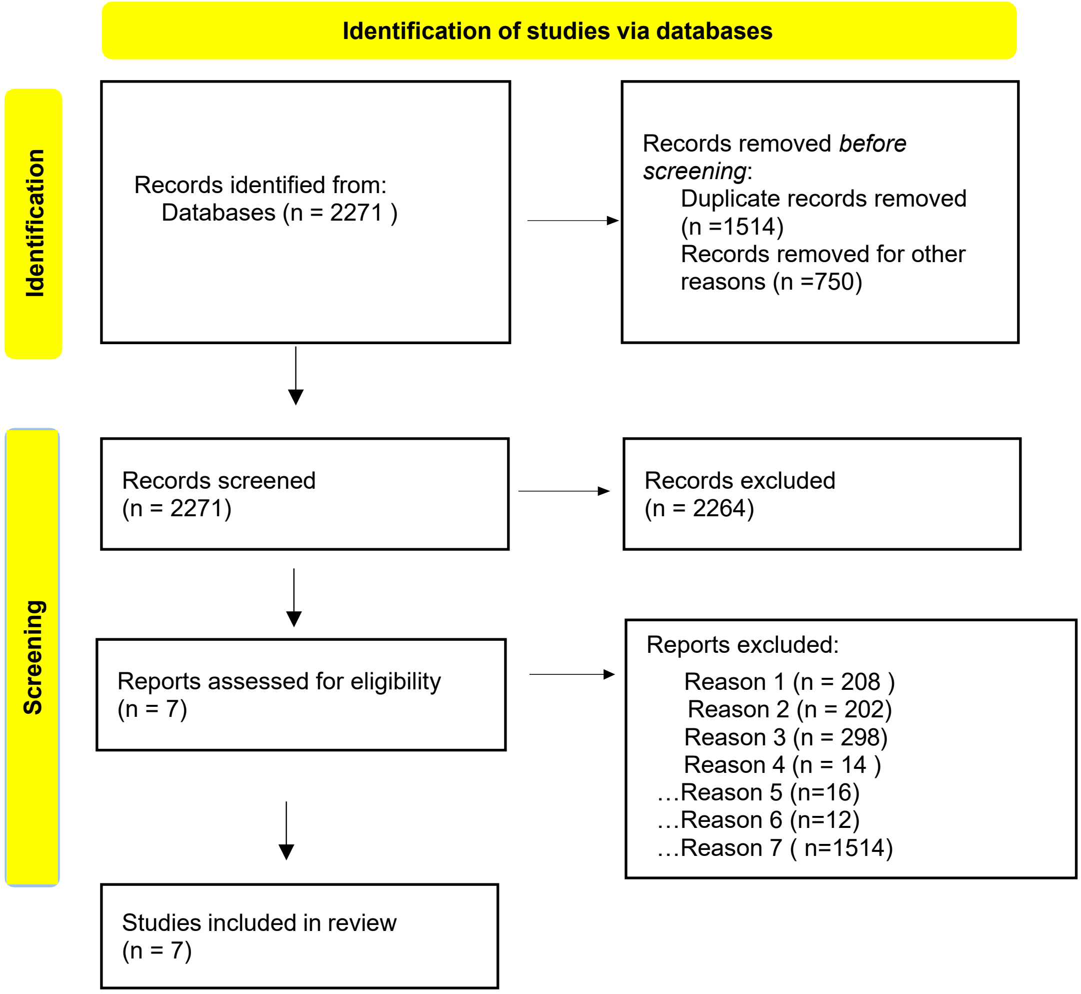

Reason 1: The study did not investigate ammonia exposure as an environmental or biological stressor.

Reason 2: The study did not assess microbiome-related outcomes, including microbial composition, dysbiosis, pathobiome dynamics, or gut–lung axis interactions.

Reason 3: The study involved irrelevant biological models or environmental contexts, including human subjects, aquatic species, soil, wastewater, or environmental microbiology.

Reason 4: The publication was a conference abstract, dataset, thesis, or another non-peer-reviewed source.

Reason 5: The study did not directly evaluate ammonia-induced alterations of the microbiome or mechanisms associated with the gut–lung axis.

Reason 6: The study was not conducted in livestock species or was considered outside the predefined scope of this review due to insufficient relevance.

Reason 7: duplicate
