# Supplementary material for: Respiratory and Gastrointestinal Microbiome Shifts in Livestock Exposed to High-Ammonia Environments: A Systematic Review
Source: Vet Sci. 2026 Sep 12;13(9):952. doi: 10.3390/vetsci13090952 (PMC13612021; doi:10.3390/vetsci13090952)
Supplement: Supplementary file 1 [file vetsci-13-00952-s001.zip › vetsci-4434675-supplementary tables.pdf]

A systematic literature search was conducted in PubMed, Web of Science Core Collection, and Scopus following the PRISMA 2020 guidelines. Database-specific search syntax was adapted according to the indexing system of each database while maintaining the same conceptual search strategy. Searches were limited to studies published within the last five years and restricted to English-language original research articles. Additional database-specific filters are detailed below.

Table S1. Database-specific search strategies used for the systematic literature search

Database	Search field	Filters applied	Boolean search strategy	Records retrieved
PubMed	Title/Abstract	Publication date: last 5 years; Full text; English; Other Animals	(ammonia[Title/Abstract] OR NH3[Title/Abstract] OR "ammonia exposure"[Title/Abstract] OR "ambient ammonia"[Title/Abstract] OR "air ammonia"[Title/Abstract]) AND (microbiome[Title/Abstract] OR microbiota[Title/Abstract] OR dysbiosis[Title/Abstract] OR pathobiome[Title/Abstract] OR "gut microbiome"[Title/Abstract] OR "intestinal microbiome"[Title/Abstract] OR "respiratory microbiome"[Title/Abstract] OR "airway microbiome"[Title/Abstract]) AND (poultry[Title/Abstract] OR broiler*[Title/Abstract] OR chicken*[Title/Abstract] OR swine[Title/Abstract] OR pig*[Title/Abstract] OR porcine[Title/Abstract] OR cattle[Title/Abstract] OR bovine[Title/Abstract]) AND (exposure[Title/Abstract] OR environment*[Title/Abstract] OR housing[Title/Abstract] OR barn*[Title/Abstract] OR air[Title/Abstract])	21
Web of Science Core Collection	Topic (TS)	Publication date: last 5 years; Research Article; English; Open Access	TS=(ammonia OR NH3 OR "ammonia exposure" OR "ambient ammonia" OR "air ammonia") AND (microbiome OR microbiota OR dysbiosis OR pathobiome OR "gut microbiome" OR "intestinal microbiome" OR "respiratory microbiome" OR "airway microbiome") AND (poultry OR broiler* OR chicken* OR swine OR pig* OR porcine OR cattle OR bovine) AND (exposure OR environment* OR housing OR barn* OR air)	73
Scopus	TITLE-ABS-KEY	Publication date: last 5 years; Research Article; English; Open Access	TITLE-ABS-KEY((ammonia OR NH3 OR "ammonia exposure" OR "ambient ammonia" OR "air ammonia") AND (microbiome OR microbiota OR dysbiosis OR pathobiome OR "gut microbiome" OR "intestinal microbiome" OR "respiratory microbiome" OR "airway microbiome") AND (poultry OR broiler* OR chicken* OR swine OR pig* OR porcine OR cattle OR bovine) AND (exposure OR environment* OR housing OR barn* OR air))	81

Table S2. SYRCLE risk-of-bias assessment of the studies included in the systematic review

**Study ID**	**Species**	**Random sequence generation**	**Baseline characteristics**	**Allocation concealment**	**Random housing**	**Blinding of caregivers/investigators**	**Random outcome assessment**	**Blinding of outcome assessment**	**Incomplete outcome data**	**Selective outcome reporting**	**Other bias**	**Overall judgement**
Study 1	Broiler chickens (*Gallus gallus domesticus*)	Low risk	Low risk	Unclear risk	Unclear risk	High risk	Low risk	Unclear risk	Low risk	Low risk	Low risk	Moderate
Study 2	Pigs (*Sus scrofa*)	Low risk	Low risk	Unclear risk	High risk	High risk	Unclear risk	Unclear risk	Low risk	Low risk	High risk	High
Study 3	Pigs (*Sus scrofa*)	Low risk	Low risk	Unclear risk	Unclear risk	High risk	Low risk	Unclear risk	Low risk	Low risk	Low risk	Moderate
Study 4	Broiler chickens (*Gallus gallus domesticus*)	Low risk	Low risk	Unclear risk	High risk	High risk	Unclear risk	Unclear risk	Low risk	Low risk	High risk	High
Study 5	Pigs (*Sus scrofa*)	Low risk	Low risk	Unclear risk	High risk	High risk	Unclear risk	Unclear risk	Low risk	Low risk	High risk	High
Study 6	Broiler chickens (*Gallus gallus domesticus*)	Low risk	Low risk	Unclear risk	High risk	High risk	Low risk	Unclear risk	Low risk	Low risk	High risk	High
Study 7	Broiler chickens (*Gallus gallus domesticus*)	Low risk	Low risk	Unclear risk	High risk	High risk	Low risk	Unclear risk	Low risk	Low risk	High risk	High
Study 8	Broiler chickens (*Gallus gallus domesticus*)	Low risk	Low risk	Unclear risk	High risk	High risk	Low risk	Unclear risk	Low risk	Low risk	High risk	High

Table S3. Base articles

No/ID	Title	Year
1	Gut microbiota dysbiosis exaggerates ammonia-induced tracheal injury Via TLR4 signaling pathway	2022
2	Exposure to the environmental pollutant ammonia causes changes in gut microbiota and inflammatory markers in fattening pigs	2021
3	Translocation of probiotics via gut–lung axis enhanced pulmonary immunity of weaned piglets exposed to low concentrations of ammonia	2024
4	Ammonia exposure induced intestinal inflammation injury mediated by intestinal microbiota in broiler chickens via TLR4/TNF-α signaling pathway	2021
5	Exposure to High Aerial Ammonia Causes Hindgut Dysbiotic Microbiota and Alterations of Microbiota-Derived Metabolites in Growing Pigs	2021
6	The alterations of tracheal microbiota and inflammation caused by different levels of ammonia exposure in broiler chickens	2021
7	Effects of ammonia on gut microbiota and growth performance of broiler chickens	2021
8	Effects of Different Ammonia Concentrations on Pulmonary Microbial Flora, Lung Tissue Mucosal Morphology, Inflammatory Cytokines, and Neurotransmitters of Broilers	2022

Table S4. Representative examples of articles excluded during title and abstract screening according to the predefined exclusion criteria.

Exclusion criterion	Representative excluded article	DOI
Not related to environmental ammonia exposure	Effects of Adding Sphingomonas Z392 to Drinking Water on Growth Performance, Intestinal Histological Structure, and Microbial Community of Broiler Chickens	10.3390/ani14131920
	Defatted Black Soldier Fly Larvae Meal in Lactating Sow and Pre-weaning Piglet Diets: Impacts on Growth Performance, Fecal Microbiota, and Metabolic Pathways	10.1163/23524588-00001120
Not related to respiratory or gastrointestinal microbiome alterations	Succession Patterns of the Bacterial Community in Poultry Litter after Bird Removal and Sodium Bisulfate Application	10.1002/jeq2.20248
	A Multifactorial Way to Assess Litter Stability and Ammonia Risk in Broiler Production: Effects Across Age (7–39 Days) and Thermal Environments	10.1080/1828051X.2025.2586295
Not conducted in targeted livestock species (poultry, swine, or cattle)	Dietary Crude Protein and Protein Solubility Manipulation Enhances Intestinal Nitrogen Absorption and Mitigates Reactive Nitrogen Emissions through Gut Microbiota and Metabolome Reprogramming in Sheep	10.1016/j.aninu.2024.04.003
	Feeding Fodder Beet (Beta vulgaris L.) versus Pasture Reduces Methane Emissions and Nitrogen Excretion in Sheep	10.1016/j.anifeedsci.2026.116804
Not relevant to the scope of the review	Feeding Amylolytic and Fibrolytic Exogenous Enzymes in Feedlot Diets: Effects on Ruminal Parameters, Nitrogen Balance and Microbial Diversity of Nellore Cattle	10.1186/s40104-025-01226-5
	Hydrogen-rich Water 400 ppb as a Potential Strategy for Improving Ruminant Nutrition and Mitigating Methane Emissions	10.1186/s12866-024-03638-1
Review article	Influence of Water Quality and Pollution on Broiler Performance, Vaccine and Antibiotic Efficiencies	10.2478/aoas-2023-0023
	Effects of Hygiene Methods on the Microbiome and Resistome of Poultry Litter	10.2478/aoas-2025-0116
Additional duplicate identified during screening	Comparative Effectiveness of Composting Sun-dried versus Insect-bioconverted Chicken Manure: Impacts on Maturity, Early Plant Development, Gaseous Emissions, and Microbial Communities	10.1016/j.wasman.2026.115453

Table S5. Representative articles excluded following full-text assessment

Exclusion criterion	n	Representative excluded article	DOI
Dietary or management interventions affecting ammonia excretion rather than environmental ammonia exposure	5	Lactobacillus-driven feed fermentation regulates microbiota metabolism and reduces odor emission from the feces of pigs	10.1128/msystems.00988-23
No mechanistic relevance regarding respiratory or gastrointestinal microbiome alterations	2	High ambient humidity aggravates ammonia-induced respiratory mucosal inflammation by eliciting Th1/Th2 imbalance and NF-κB pathway activation in laying hens	10.1016/j.psj.2022.102028
Total excluded after full-text assessment	7	—	—
Studies included in the systematic review	8	—	—
